# Activity-dependent plasticity of electrical synapses: increasing evidence for its presence and functional roles in the mammalian brain

**DOI:** 10.1186/s12860-016-0090-z

**Published:** 2016-05-24

**Authors:** Julie S. Haas, Corey M. Greenwald, Alberto E. Pereda

**Affiliations:** Department of Biological Sciences, Lehigh University, Bethlehem, PA 18015 USA; Dominick P. Purpura Department of Neuroscience, Albert Einstein College of Medicine, Bronx, New York, NY 10461 USA

## Abstract

Gap junctions mediate electrical synaptic transmission between neurons. While the actions of neurotransmitter modulators on the conductance of gap junctions have been extensively documented, increasing evidence indicates they can also be influenced by the ongoing activity of neural networks, in most cases via local interactions with nearby glutamatergic synapses. We review here early evidence for the existence of activity-dependent regulatory mechanisms as well recent examples reported in mammalian brain. The ubiquitous distribution of both neuronal connexins and the molecules involved suggest this phenomenon is widespread and represents a property of electrical transmission in general.

## Background

The functions of electrical synapses have recently been of increasing interest within the neuroscience community. Electrical transmission is supported by gap junctions, structures that are formed by plaques of paired and docked connexin-formed hemichannel pores in apposed neuronal cell membranes. These channels form a physical connection between cells, allowing ionic current to flow directly between coupled neurons. Electrical synapses in most mature mammalian neuronal systems are composed of connexin36 (Cx36) [1–3] and connect, amongst many cell types, GABAergic neurons of similar biochemical subtype [4, 5], which are widespread. Both gap junctions between neurons and electrical transmission have been identified in a still-increasing number of systems and brain areas, reinforcing the notion that electrical synapses contribute vitally to information processing across the brain.

Like chemical synapses, electrical synapses can vary their gain [6, 7]. Modifications of synaptic strength are thought to underlie important functional processes, including learning and memory [8, 9]. Modification of the strength of electrical synapses was initially reported as a result of the action of neurotransmitter modulators [6, 10], such as dopamine [11], which also modulates chemical synapses [12] and neuronal excitability [13].

More recent evidence indicates that the strength of electrical synapses is influenced by ongoing activity in neural networks, via interactions with chemical synapses [14]. ‘Activity-dependent plasticity’ of electrical transmission was initially reported in fish, at auditory nerve mixed synapses on the Mauthner cells [15]. Here we review mammalian structures in which activity-dependent plasticity of electrical transmission has been demonstrated: the retina, the thalamic reticular nucleus (TRN) and the inferior olive, as well early evidence in the anterior hypothalamus. Both the widespread distribution of the involved molecules and common regulatory mechanisms suggest that plasticity is an essential and ubiquitous property of electrical transmission in the mammalian brain.

## Mixed synapses on the Mauthner cells

Mauthner cells mediate escape reflex in fish (and amphibian tadpoles) and receive auditory input from the nerve afferents that terminate as ‘club endings’, a synapse that combines chemical and electrical transmission [16–18]. Electrical synapses between VIIIth-nerve auditory afferents and Mauthner cells are composed of hemichannels formed by two teleost homologs of the mammalian Cx36: Cx35 at presynaptic hemiplaque sides, and Cx34.7 at postsynaptic hemiplaques, form heterotypic gap junctions [19]. This molecular asymmetry is mirrored by functional asymmetry, averaging a 4-fold differential of electrical transmission in favor of the presynaptic club ending, also enhancing the excitability of neighboring club endings onto the same Mauthner cell.

Several types of stimuli have been shown to induce plasticity of the electrical component within these synapses. Discontinuous bursts of tetanizing stimulation of the VIIIth nerve leads to long-term potentiation of the electrical component of the EPSP [15, 20, 21] with a parallel increase in the chemical excitatory component of the EPSP. This form of plasticity depends on calcium (Ca^2+^) increase, which activates a Ca^2+^/calmodulin-dependent kinase (CaMKII) [22], and involves nearby NMDARs [23]. Brief continuous high-frequency stimulation of the VIIIth nerve also leads to potentiation, through mGluR1-dependent endocannabinoid production and release of dopamine, which in turn acts postsynaptically via activation of D1/5 receptors and cAMP-dependent protein kinase A (PKA) [24]. Thus, both forms of activity-dependent potentiation of the Mauthner synapse depend on the activation of glutamate receptors localized in the same contact. In addition, activation of opioid receptors was shown to lead to long-term enhancement of electrical (and glutamatergic) transmission at Mauthner cells. Although no specific forms of neuronal activity patterns have been so far identified for this mechanism it also requires as in the case of endocannabinoids activation of dopamine D1/5 receptors and postsynaptic PKA [25], suggesting the existence of interactions between both potentiating mechanisms.

Together, these results indicate a high degree of sensitivity of Mauthner electrical synapses to neuronal activity and signaling. While the sensory stimulus that triggers an escape response is likely multimodal, and combines vestibular and lateral line information [26, 27], the plasticity of the electrical component of the synapse is likely to render the Mauthner cell more responsive to afferent stimuli both from the VIIIth nerve and, potentially, from other afferents. Enhanced electrical coupling would feed the depolarization produced by other active afferents back to neighboring inactive synapses, increase their excitability and promote cooperativity between afferents to the Mauthner cell [28, 29]. The phenomenon of lateral excitation is also supported by the functional asymmetry of this synapse, which favors electrical transmission in the antidromic direction (from the Mauthner cell to the presynaptic afferents) [19]. While reliable depressing stimuli has yet to be identified (but see [30]), we speculate that plasticity in this synapse is an important component in online adjustments in the overall sensitivity of the Mauthner cell, and the associated escape reflex, to afferent sensory information.

## Retina

The retina has provided the earliest and more numerous examples of regulation of electrical synapses by neurotransmitter modulators. Electrical synapses appear widely across the retina, which contains layered structures of cells that are conserved between fish and mammalian retinas. Variations in electrical synapse strength contribute to tuning the sensitivity of retinal circuits for transitioning between nighttime and daytime visual tasks. In the outer layer, coupling connects both functionally similar (e.g. cone-cone) and dissimilar (rod-cone) types of photoreceptors. Dispersion of voltage signals between cells helps to suppress voltage noise in individual cells. Moreover, the rod pathway informs the cone pathway of light levels at the upper end of its dynamic range. In bright light, rod-cone coupling is reduced and rod input is effectively eliminated from the cone pathway by a dopamine-mediated circadian mechanism [31, 32]. In goldfish, activation of dopamine receptors in the daylight, and adenosine by night, regulates PKA-mediated phosphorylation of Cx36 in mouse cone cells [33]. Modifications in coupling have also been demonstrated in the inner nuclear layer. Here, dopamine regulates coupling between horizontal cells, which express gap junctions based on the connexins Cx57 or Cx50 in mammals and several homologous connexins in fish, by modulating open probability [11, 34]. Retinal AII amacrine cells are extensively coupled with other AII amacrine cells, and with cone bipolar cells.

In addition to the action of neuromodulators, recent data indicates that mammalian retinal electrical synapses are also sensitive to activity of glutamatergic synapses. That is, AII amacrine cell synapses undergo changes in electrical coupling driven by light adaptation [35], in which glutamate spillover from bipolar cell spiking activity produces enhancement of coupling via activation of NMDA receptors, CaMKII, and phosphorylation of Cx36 [36]. Retinal electrical synapses are but one of many mechanisms that contribute to the visual adaptation processes necessary to meaningfully handle overall changes in light intensity over a billion-fold range. The retina contains many embedded microcircuits that process different specific features of the visual world in parallel. Activity-driven glutamate-dependent electrical synapse plasticity may tune and/or isolate these feature-selective pathways within that circuit.

## Inferior olive

The olivo-cerebellar network provides the timing signals necessary for precise coordination of motor actions, and for non-motor and cognitive tasks. Within the network, neurons of the inferior olive (IO) provide the powerful excitatory climbing fiber input to Purkinje neurons. The clock of the timing signals in the cerebellum is thought to be provided by subthreshold 5–10 Hz oscillations produced by intrinsic mechanisms within neurons of the IO [37–39]. Electrical coupling between IO cells is thought to synchronize their sparse spikes [37, 40, 41].

IO neurons are also influenced by GABAergic input from the cerebellar nuclei in IO glomeruli, where dendrites of neighboring IO neurons are connected via gap junctions [42] that are thus electrotonically distant from the somatic integrator. [For this reason, coupling coefficients measured between somas in IO neurons are typically weak [40]]. Rodolfo Llinás and colleagues hypothesized that electrical coupling between IO neurons is transiently modulated by synaptic inputs that act as a shunt between the GJs and soma, which results in an apparent depression of electrical synapse strength [43]. This mechanism has been recently directly confirmed by optogenetic activation of GABAergic input from the cerebellar nuclei that caused a transient decrease in electrical coupling strength between olivary cells [44].

Another recent set of work has investigated long-term electrical synaptic plasticity coincident with glutamatergic activity in this structure. Long-term potentiation of electrical synapses between pairs of IO cells results from high-frequency stimulation or NMDA application, an effect dependent on intracellular Ca^2+^ and CaMKII activity [45]. Conversely, lower-frequency stimulation (1 Hz) of adjacent white matter leads to depression of coupling, an effect also mediated via activation of NMDA receptors [46]. The involvement of NMDA receptors in triggering both activity-dependent potentiation and depression of synaptic transmission was previously reported for chemical synapses [47]. NMDAR-dependent bi-directional plasticity of electrical transmission in the IO is likely related to differences in the induction protocols or small variations in the experimental conditions.

Coupling among IO neurons is highly variable, heterogeneity that was proposed to result from short-term activity-dependent plasticity at individual glomeruli [48]. Coupling was also reported to be asymmetrical [40], suggesting that substructures or microcircuits within IO circuits, defined by coupling, are formed and adjusted by ongoing changes in the strength of electrical synapses. Indeed, plasticity of electrical synapses has been proposed as a mechanism whereby motor learning and increased precision of timing is accomplished by gradual reduction of coupling strength in small subsets of IO neurons [49].

## Thalamic reticular nucleus

The thalamic reticular nucleus (TRN) forms a dorsolateral shell around the thalamus proper. This structure primarily receives glutamatergic input from the corticothalamic and thalamocortical axons, and projects inhibitory GABAergic synapses to neurons of the thalamus [50, 51]. Together, TRN neurons act to gate information between the cortex and the thalamus [52, 53]. This “spotlight” has been proposed to focus cortical attention on important stimuli though coordinated inhibition of distractions. Within the TRN, neurons are densely and strongly coupled [54, 55] by Cx36-based GJs [1].

Two forms of electrical synapse depression have emerged in the TRN. First, tetanic stimulation of cortical afferents to the TRN result in an glutamate-mediated long-term depression of electrical synapses [56]; activation of Group I mGluRs induces long-term depression, while activation of Group II receptors induces long-term potentiation, of electrical synapses in a competing pathway through cAMP and PKA [57].

Synchronous low-frequency bursting activity induced in coupled TRN neurons also leads to depression of the electrical synapses between them [58] via a yet-undetermined mechanism. Bursting activity in TRN neurons is a prominent component of both sleep spindle rhythms [59, 60] and the sharp wave discharges that characterize absence seizures [61, 62]. Depression resulting from induced paired bursting in TRN neurons offers the most specific evidence to date that ongoing glutamate-driven physiologically patterned activity can modify electrical synapse strength.

In the TRN, depression of electrical synapses generally acts to desynchronize the spiking of active neurons. The TRN is a regulator of thalamocortical spindle rhythms [63]; plasticity of electrical synapses may be a regulatory mechanism for maintaining physiological levels of synchrony, by uncoupling neurons that are overly synchronized. Interestingly, transmission at electrical synapses in the TRN is asymmetrical [64] and, further, activity-induced depression of those asymmetrical synapses acts to increase that inequality of transmission [58], suggesting that plasticity could play a physiological role by selectively enhancing or diminishing asymmetry of transmission, and thereby the direction of information flow across the TRN. A thought-provoking possibility is that depression could occur between active sensory subsectors within the TRN, and results in dynamic isolation of one sensory modality within the nucleus from others. Alternatively, activity-dependent plasticity of electrical synapses may desynchronize neurons within a sensory subsector that are active in response to specific stimuli.

## Anterior hypothalamus

The established role of glutamatergic transmission in promoting activity-dependent plasticity of electrical transmission is consistent with earlier reports in hypothalamus. Glenn Hatton and colleagues [65, 66] reported that electrical stimulation of the lateral olfactory tract led to an increase in dye coupling between neurons of the supraoptic nucleus. Remarkably, this increase was observed in lactating but not virgin or male rats [66]. Although the involvement of glutamate receptors was not pharmacologically tested in those experiments, the lateral olfactory tract is known to carry monosynaptic projections from mitral cells in the olfactory bulb which are glutamatergic in nature [67, 68] suggesting the involvement of their receptors in the induction of plastic changes. In retrospect this report could be then considered the first evidence of activity-dependent increase in coupling resulting from synaptic activation in the mammalian CNS. Subsequent results in the suprachiasmatic nucleus of the hypothalamus have shown a decrease in dye coupling resulting from TTX blockade of activity [69], while application of vasoactive intestinal peptide increases spiking activity [70] and coupling coefficients [71].

From the functional point of view, these data suggest that coupling increases following strong sensory inputs associated with behavior that result in increased hormone or peptide release in response to other incoming stimuli.

## Conclusions

**Fig. 1:**
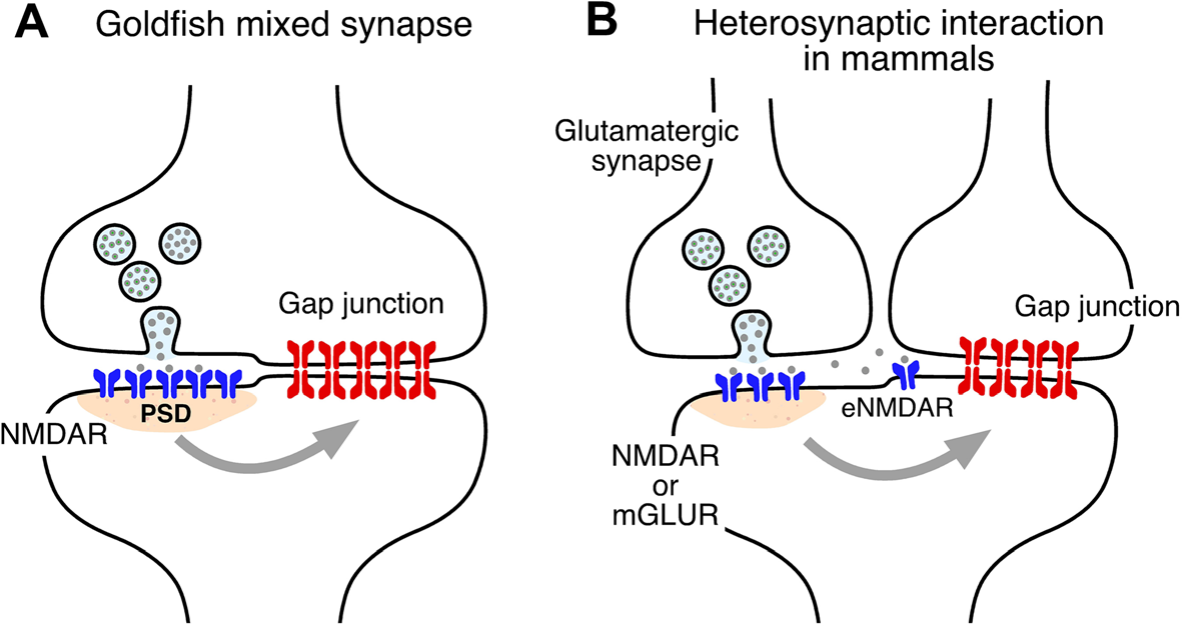
Interactions between glutamatergic and electrical synapses that leads to activity-dependent potentiation of electrical transmission. **a** At goldfish mixed synapses the activity of co-existing glutamatergic synapses leads to activation of NMDARs which initiates changes in electrical (and chemical) transmission [the second form of activity-dependent potantiation involvimg mGluRs, endocannabinoids and dopamine is not represented]. **b**, At mammals, activation of mGluRs or NMDARs, including extrasynaptic NMDARs (eNMDAR), leads to changes in electrical transmission

**Fig. 2:**
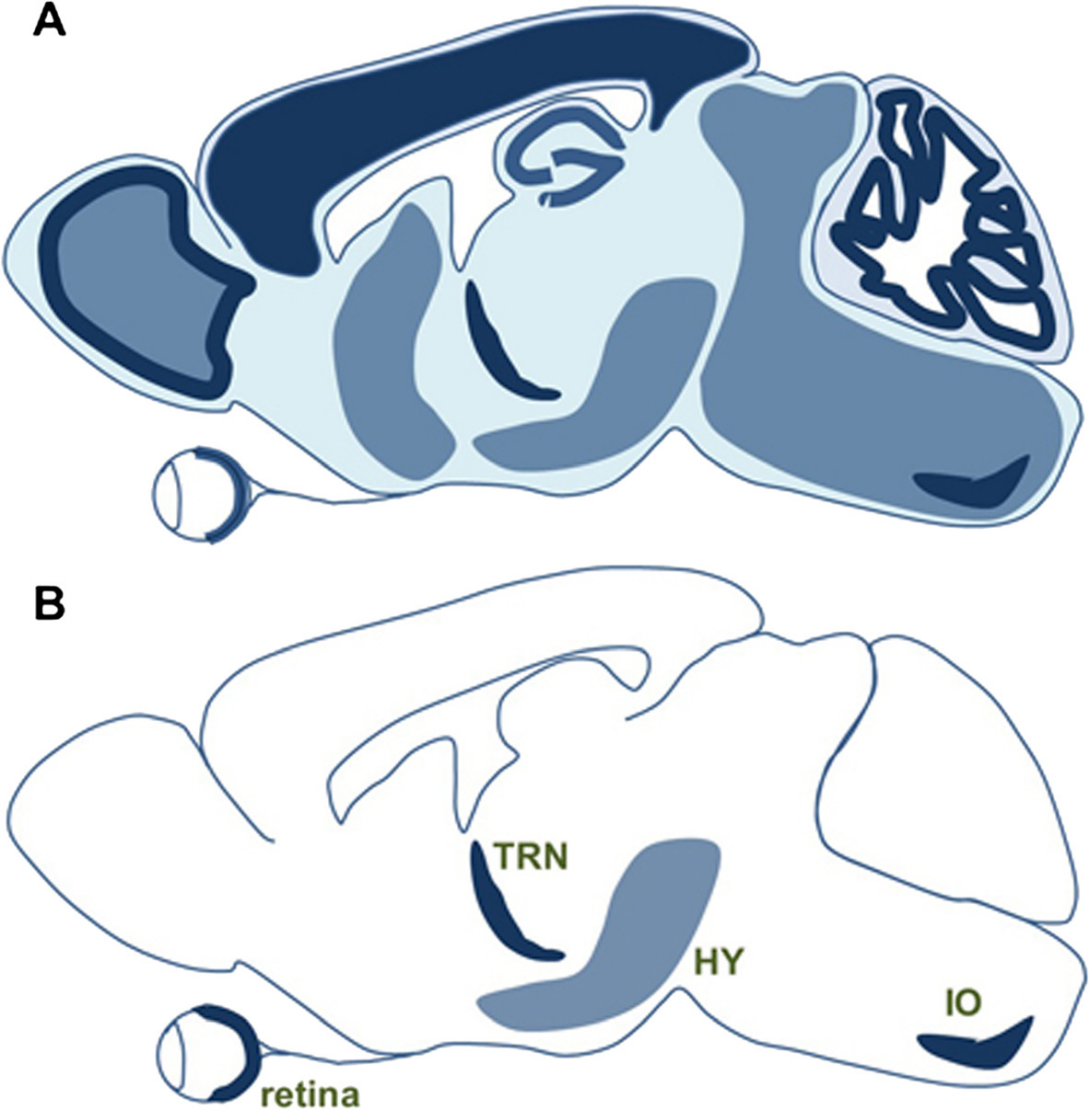
Activity-dependent plasticity of electrical transmission across the mammalian brain. **a** Both Cx36 and GluA (a marker of glutamatergic transmission) are expressed (light filled background) broadly across the rat brain. Darker areas represent areas with higher Cx36 expression. **b** Activity-dependent modification of electrical transmission has been currently reported in only four areas (TRN, thalamic reticular nucleus; HY, hypothalamus; IO, inferior olive)

While electrical synapses are known to be the target of the neuromodulatory transmitters, increasing evidence indicates that they are profoundly influenced by the activity of the networks in which they are embedded. This activity-dependent plasticity of electrical transmission has been shown to rely, so far, on interactions with nearby chemical synapses via activation of glutamate receptors. Originally identified in fish (Fig. 1a), this form of interaction between chemical and electrical synapses has been shown to occur in at least four different mammalian structures (Fig. 1b). The widespread distribution of Cx36 and glutamate transmission in the mammalian CNS (Fig. 2a) suggests this interaction might be common, while these examples (Fig. 2b) represent just the tip of the iceberg. Furthermore, similar to goldfish mixed synapses, glutamate receptor-containing postsynaptic densities have been shown to be located nearby Cx36-containg gap junctions in various structures of the mammalian brain [14], extrasynaptic NMDA receptors were identified in close proximity to Cx36-containing gap junctions [45, 72], and new anatomical evidence points towards the possibility of mixed synapses in the mammalian auditory brainstem [73]. Although is not the topic of this article, interactions between glutamate receptors and electrical synapses are not restricted to the adult brain. Activation of Group II mGluRs at early developmental stages leads to an increase in Cx36 expression, whereas activation of NMDARs leads to its decrease at late developmental stages (for review see [74]).

The distinction between neuromodulator-dependent and activity-dependent plasticity is somewhat arbitrary, and both regulatory processes are likely to interact. For instance, a form of activity-dependent potentiation at goldfish mixed synapses was shown to require activation of mGluR1 receptors that, by promoting the release of endocannabinoids, led to the release of dopamine from nearby varicose terminals, which in turn triggers potentiation via a postsynaptic mechanism [24, 74].

Beyond interactions with chemical synapses, activity-dependent plasticity of electrical transmission is likely to occur via other mechanisms. Interestingly, a recent report indicates that the induction of gap junction plasticity occurred in the absence of chemical synaptic stimulation and was driven by bursting cellular activity [58], suggesting that plastic changes can indeed occur in the absence of interactions with nearby glutamate receptors, and are possibly initiated by ion influx during spiking activity. Finally, could the activity of electrical synapses themselves lead to their potentiation? Although so far there are no examples of this exciting possibility, gap junction channels are part of multiprotein complexes [75] and are known to be associated to a range of signaling molecules [76] including transcription factors [77, 78], suggesting they might be endowed with the machinery required to induce plasticity.
